# Maternal Emulsifier P80 Intake Induces Gut Dysbiosis in Offspring and Increases Their Susceptibility to Colitis in Adulthood

**DOI:** 10.1128/mSystems.01337-20

**Published:** 2021-03-16

**Authors:** Ge Jin, Qiang Tang, Jiaheng Ma, Xiang Liu, Bingqian Zhou, Yue Sun, Xiaoqi Pang, Zixuan Guo, Runxiang Xie, Tianyu Liu, Bangmao Wang, Hailong Cao

**Affiliations:** a Department of Gastroenterology and Hepatology, General Hospital, Tianjin Medical University, Tianjin, China; b Tianjin Institute of Digestive Diseases, Tianjin Key Laboratory of Digestive Diseases, Tianjin, China; c Department of Gastroenterology and Hepatology, Tianjin Union Medical Center, Tianjin, China; Southern Medical University

**Keywords:** early life, emulsifier, intestinal development, gut microbiota, colitis

## Abstract

Early life events can lead to multiple diseases in adulthood. Previous studies suggested that polysorbate 80 (P80) as a widely used emulsifier in pharmaceutical formulations and food industries could impair the intestinal barrier. However, whether maternal P80 (MP80) exposure could affect the long-term health of offspring remains unknown. In this study, we found that maternal P80 intake could retard intestinal development, disrupt the intestinal barrier, and cause low-grade intestinal inflammation in 3-week-old offspring. 16S rRNA sequencing and correlation analysis revealed that *Mucispirillum*, *Clostridium XI*, and *Parabacteroides*, which positively correlated with intestinal proliferation and differentiation, were decreased in the maternal P80 group. Interestingly, the increase in some harmful bacteria, including *Proteobacteria*, *Helicobacteraceae, Campylobacterales*, and *Desulfovibrionales*, persisted from the weaning period to adulthood (3 to 8 weeks). Furthermore, a fecal microbiota transplantation assay showed that the mice gavaged with feces from 3-week-old offspring of the MP80 group presented more severe intestinal inflammation and barrier disruption than the mice that received feces from the offspring of the control group. Finally, maternal P80 intake remarkably aggravated the structural disorder of intestinal crypt, increased proinflammatory factors, and exacerbated dextran sulfate sodium (DSS)-induced colitis in adulthood. Conclusively, maternal P80 intake could induce gut dysbiosis and promote colitis susceptibility in adulthood. This study provides new insights into the prevention of inflammatory bowel disease (IBD).

**IMPORTANCE** The main findings of this research showed that maternal P80 intake could disrupt the intestinal barrier, induce gut dysbiosis, and promote colitis susceptibility in adulthood. This study will enhance understanding of the prevention of IBD.

## INTRODUCTION

Polysorbate 80 (P80), also called Tween 80, is a synthetic nonionic substance that consists of the fatty acid esters of polyoxyethylene sorbitan. P80 has lipophilic and hydrophilic moieties that can prevent surface adsorption and protein aggregation. Hence, P80 is ubiquitously applied to the manufacture of food, drugs, and personal care products as an emulsifier (1–3). According to data revealed by the Food and Drug Administration (FDA), the mean exposure to P80 of American citizens from 2003 to 2010 was 8 mg/kg body weight per day (4). Although the FDA insists that the tiny dosage of P80 should not raise a safety concern (4), many studies are still questioning the safety of P80. Interestingly, B. Chassaing uncovered that P80 could erode intestinal mucus and elicit gut microbiota dysbiosis, which leads to metabolic syndrome and low-grade inflammation (5). P80 exposure could also impact the growth rate of bacteria and subsequently perturb biofilm formation (6).

Inflammatory bowel disease (IBD) has become a global challenge in the 21st century. The incidence of IBD in Asia and South America has ascended dramatically from the 1990 until now, along with urbanization and industrialization; hence, environmental determinants may play a crucial role in IBD (7–11). IBD is characterized by chronic and recurrent inflammation in the gut and is widely believed to be a multifactorial disease that involves genetic variation, environmental exposure, intestinal barrier dysfunction, gut dysbiosis, and aberrant immune responses (12–14). The onset of chronic inflammation in genetically susceptible individuals may be triggered by the interplay between environmental factors and the innate immune system mediated by dysbiosis via an impaired intestinal barrier (12). Dietary factors, which directly alter the composition and metabolism of gut microbiota, are one of the crucial environmental determinants of IBD (7–9, 14, 15).

The evolution of an infant’s gut microbiota composition toward an adult-like pattern is accomplished during the first 3 years after birth (16). This critical period, which is of great importance to an individual’s health, metabolism, and ontogeny, is defined as the early life stage (17–20). Vertical microbiota transmission from mothers to their infants may occur via feces, vaginal delivery, skin, and breastfeeding (21–28), and maternal intestinal microorganisms exert a major influence on the offspring’s microbiome (29). Validation with several animal models indicated that eliciting maternal gut dysbiosis during gestation and lactation could interfere with the offspring’s microbial composition, ontogeny, metabolism, and immune system maturation (30–34). Epidemiological studies have uncovered the correlation between the disruption of early life gut microbiota colonization and the incidence of multiple diseases, such as asthma, allergy, and eczema (35–41). However, no research has been reported on the association between the susceptibility to adult colitis and P80 intake in early life.

The current study aimed to investigate whether early life exposure to P80 would perturb individuals’ gut microbiota and alter IBD susceptibility in adulthood. We found that maternal P80 (MP80) intake inhibited intestinal development and cell proliferation, damaged the mucosal barrier, and induced low-grade inflammation and gut dysbiosis. Interestingly, gut dysbiosis that persisted from 3 weeks to 8 weeks might result in susceptibility to dextran sulfate sodium (DSS)-induced colitis in adulthood. The findings will provide new insights into IBD prevention.

## RESULTS

### Maternal P80 intake perturbed intestinal development of offspring.

**FIG 1:**
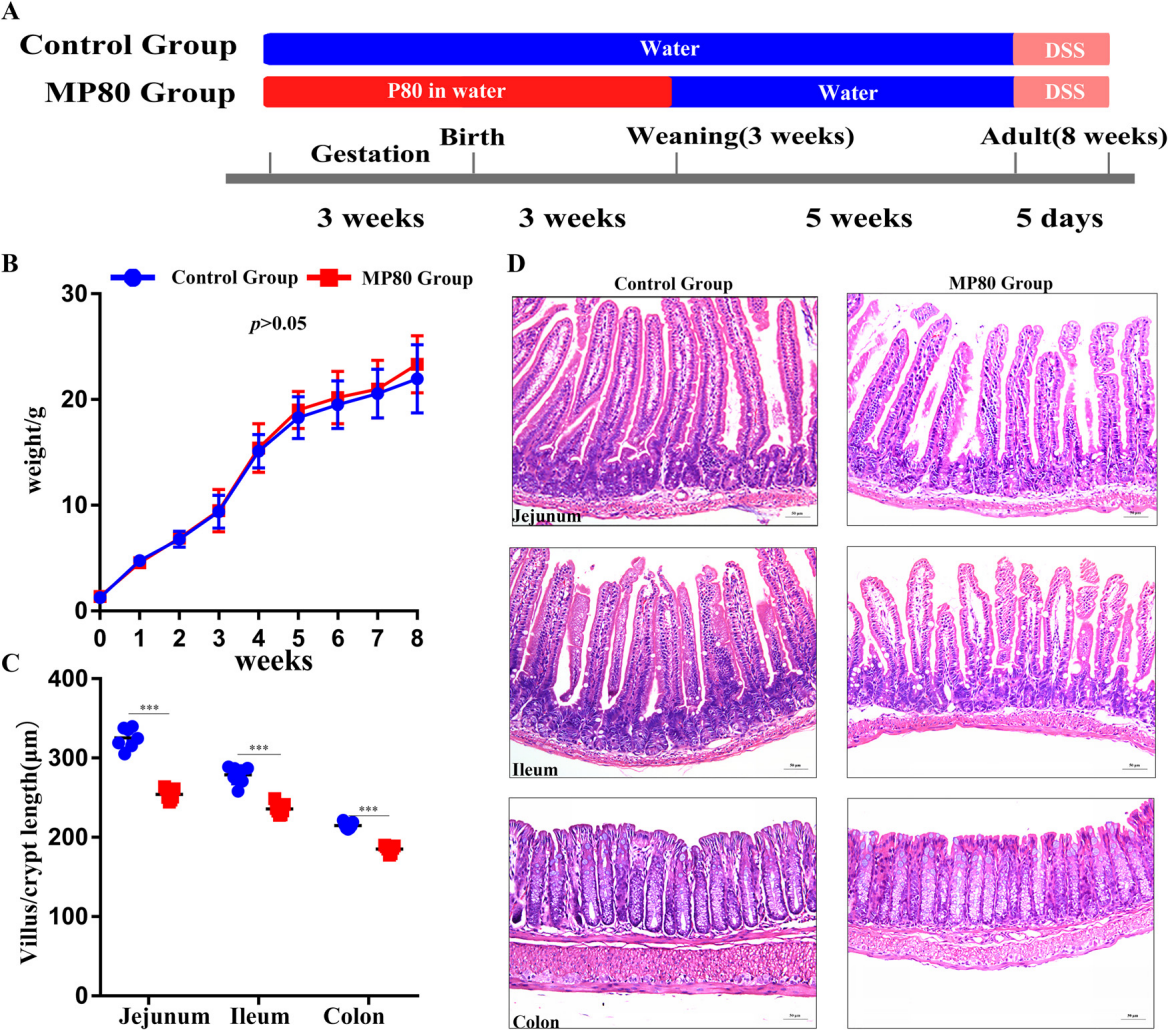
Maternal P80 intake perturbed the intestinal development of 3-week-old offspring. (A) Experimental process. (B) Body weight of the mice until 8 weeks postpartum. (C) The lengths of 100 villi and the depths of 100 crypts in each offspring. (D) Intestinal tissue stained with H&E. P80, Polysorbate 80; MP80, maternal P80. *n* = 17 in each group. Scale bars, 50 μm. ***, *P* < 0.001. *P* values of >0.05 are nonsignificant.

The experimental process is shown in Fig. 1A. Diet and water consumption during pregnancy between the MP80 group and control group had no remarkable difference. The weight of offspring in the MP80 group (10 females and nine males) did not differ substantially from those in the control group (10 females and nine males) (Fig. 1B). At 3 weeks, seven mice from both groups were randomly selected and sacrificed (MP80 group, five females and two males; control group, four females and three males). Intestinal villi play a critical role in nutrient absorption, and the length of the villi can reflect absorption area and capacity. Hematoxylin and eosin (H&E) staining showed that the length of villi in the small intestines and the depth of crypts in the colon of 3-week-old offspring in the MP80 group were remarkably decreased compared with those in the control group; thus, P80 intake in early life could inhibit ontogeny of gut (Fig. 1C and D).

**FIG 2:**
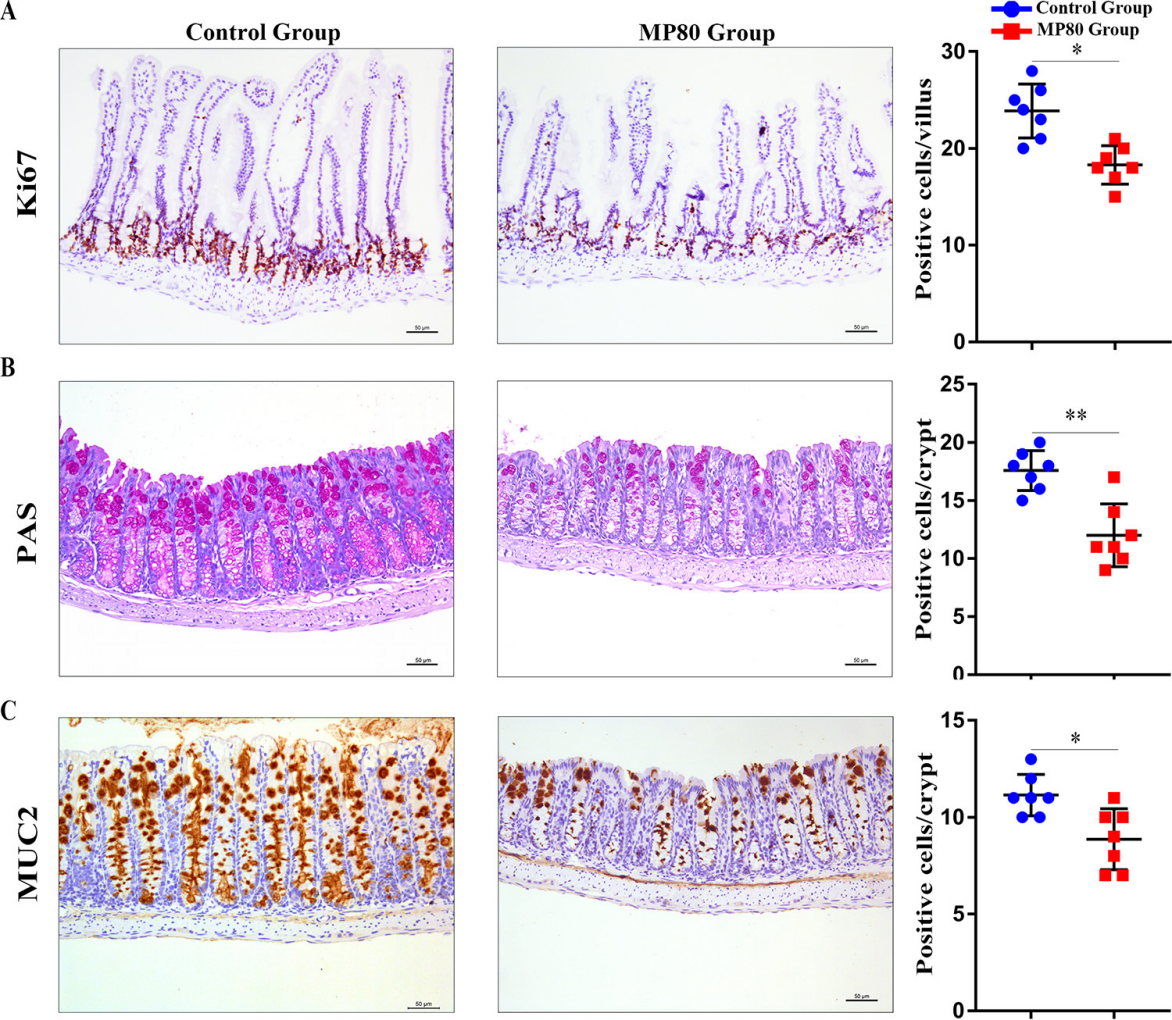
Maternal P80 intake interrupted intestinal proliferation and differentiation in 3-week-old offspring mice. (A) Intestinal proliferation as assessed by Ki67 immunohistochemical staining. (B) Quantification of goblet cells by PAS staining. (C) Product of MUC2. MP80, maternal P80. *n* = 7 in each group. Scale bars, 50 μm. *, *P* < 0.05; **, *P* < 0.01.

In rodents, the functional maturation of the gastrointestinal tract usually occurs at 3 weeks after birth and could be estimated by cell proliferation, differentiation, and migration. The number of Ki67 antibody-positive cells in ileum (Fig. 2A) decreased substantially in the pups of the MP80 group. Periodic acid-Schiff (PAS) staining showed that the number of goblet cells in colon remarkably decreased in the pups of the MP80 group (Fig. 2B). Therefore, maternal P80 intake in early life causes stunting in the offspring but does not cause obesity.

### Maternal P80 intake disrupted the intestinal barrier and promoted intestinal low-grade inflammation.

**FIG 3:**
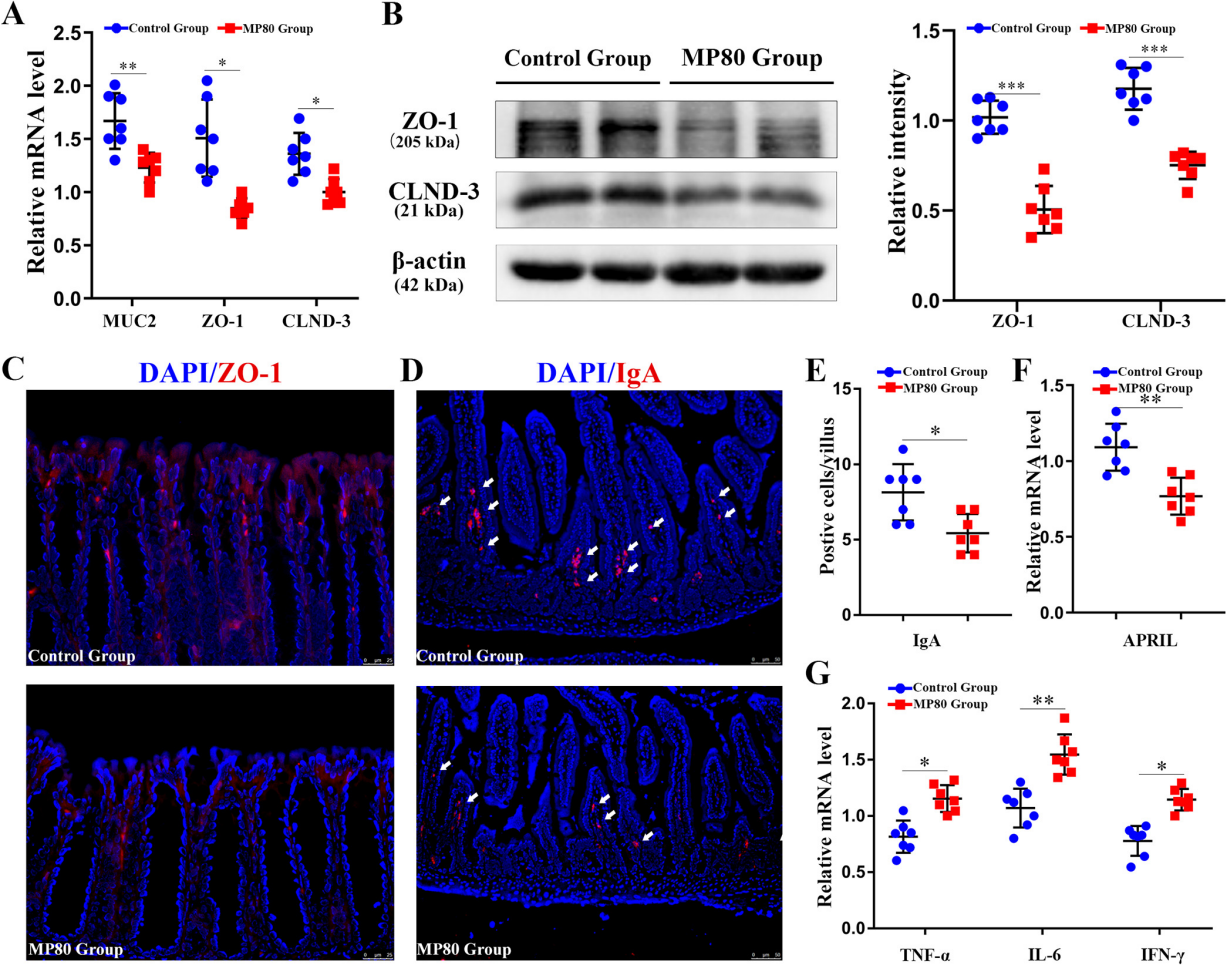
Maternal P80 intake disrupted the intestinal barrier and induced intestinal low-grade inflammation in 3-week-old offspring mice. (A) RNA expression of MUC2, ZO-1, and CLND-3 as quantified by RT-PCR. (B) Protein levels of ZO-1 and CLND-3 in the colonic tissue from 3-week-old offspring mice as detected by Western blotting and relative intensity as quantified by ImageJ software. (C and D) ZO-1 (C) and IgA (D) distributions as visualized by immunofluorescence. (E) IgA-positive cells in 100 villi. (F) Relative mRNA expression of APRIL. (G) Relative mRNA expression of proinflammatory cytokines, including TNF-α, IL-6, and IFN-γ, as detected by RT-PCR. *n* = 7 in each group. MP80, maternal P80. *, *P* < 0.05; **, *P* < 0.01; ***, *P* < 0.001.

Intestinal barrier defect, a critical trigger of inflammation, results in many diseases, including IBD (42, 43). Mucin-2 (MUC2) is the main mucin produced by goblet cells and forms the intestinal mucus barrier. The number of positive cells in colon and the mRNA level of MUC2 were considerably decreased in the pups of the MP80 group (Fig. 2C and 3A). We investigated the impacts of MP80 intake on the intestinal tight junction (TJ) of 3-week-old pups. The mRNA levels of zonula occludens 1 (ZO-1) and claudin-3 (CLND-3) were remarkably lower in the MP80 group than the control group (Fig. 3A). The protein expression of ZO-1 and CLND-3 in the colonic tissues was consistent with the PCR results (Fig. 3B). Furthermore, maternal P80 intake could perturb the integrity of ZO-1 in the colonic membrane, as visualized by immunofluorescence staining (Fig. 3C).

Immunoglobulin A (IgA), an important antibody in the gut, plays a crucial role in the maintenance of intestinal immunological function. Secretory IgA (sIgA) also exerts a considerable influence on the regulation of gut homeostasis. sIgA was remarkably decreased in the MP80 group (Fig. 3D and E). Moreover, the relative mRNA level of a proliferation-inducing ligand (APRIL) that is associated with the production of sIgA in IgA-secreting plasma cells was substantially lower in the MP80 group (Fig. 3F).

No apparent microscopic intestinal inflammation in the offspring in both groups was observed by H&E staining. However, the relative mRNA levels of proinflammatory cytokines, including tumor necrosis factor alpha (TNF-α), interleukin-6 (IL-6), and gamma interferon (IFN-γ), were remarkably higher in the MP80 group (Fig. 3G). Hence, MP80 intake could promote intestinal low-grade inflammation in the offspring.

### Gut microbiota from the MP80 group impaired intestinal barrier function and induced low-grade inflammation.

**FIG 4:**
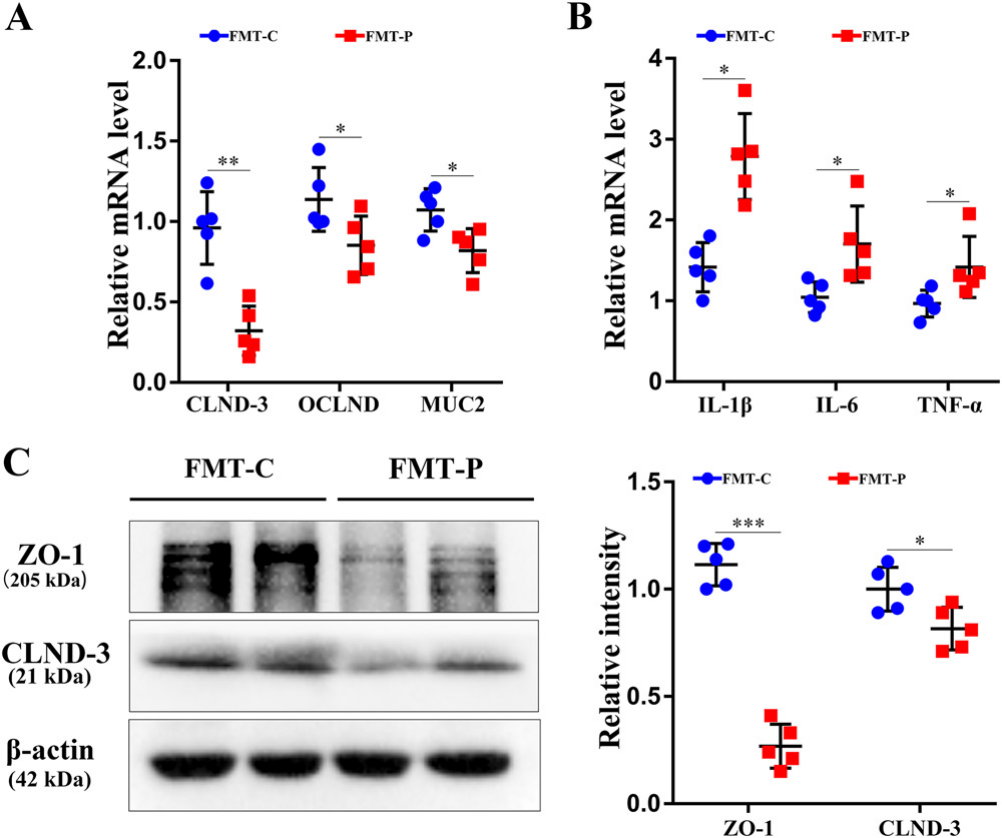
Gut microbiota from the MP80 group impaired intestinal barrier function and induced low-grade inflammation. (A) Relative mRNA levels of CLND-3, OCLN, and MUC2 in the FMT-P and FMT-C groups. (B) Expression levels of TNF-α, IL-6, and IL-1β. (C) Protein expression of ZO-1 and CLND-3 as detected by Western blotting and relative intensity as quantified by ImageJ software. *n* = 5 in each group. FMT-P group, fecal microbiota transplantation P80 group; FMT-C group, fecal microbiota transplantation control group. *, *P* < 0.05; **, *P* < 0.01; ***, *P* < 0.001.

Interestingly, we found that the relative mRNA levels of CLND-3, OCLN, and MUC2 in the FMT-P group (fecal microbiota transplantation [FMT] P80 group, gavaged with fecal samples from the MP80 group) were lower than those of the FMT-C group (FMT control group, gavaged with fecal samples from the control group) (Fig. 4A). The protein expression of ZO-1 and CLND-3 was lower in the colonic tissues of the FMT-P group (Fig. 4C). Moreover, the relative mRNA levels of proinflammatory cytokines, including TNF-α, IL-6, and IL-1β, were remarkably higher in the FMT-P group (Fig. 4B). These results indicated that gut microbiota from MP80 group impaired intestinal barrier function and induced low-grade inflammation.

### Maternal P80 intake induced gut microbiota dysbiosis in 3-week-old offspring that continued until 8 weeks old.

Gut microbiota is separated from the colonic epithelium by a healthy mucus layer, which limits mucosal inflammatory response. In the present study, we found that the intestinal barrier was disrupted because of maternal P80 intake (Fig. 2C). Then, pathogenic bacteria became a driving force for the induction of inflammation.

**FIG 5:**
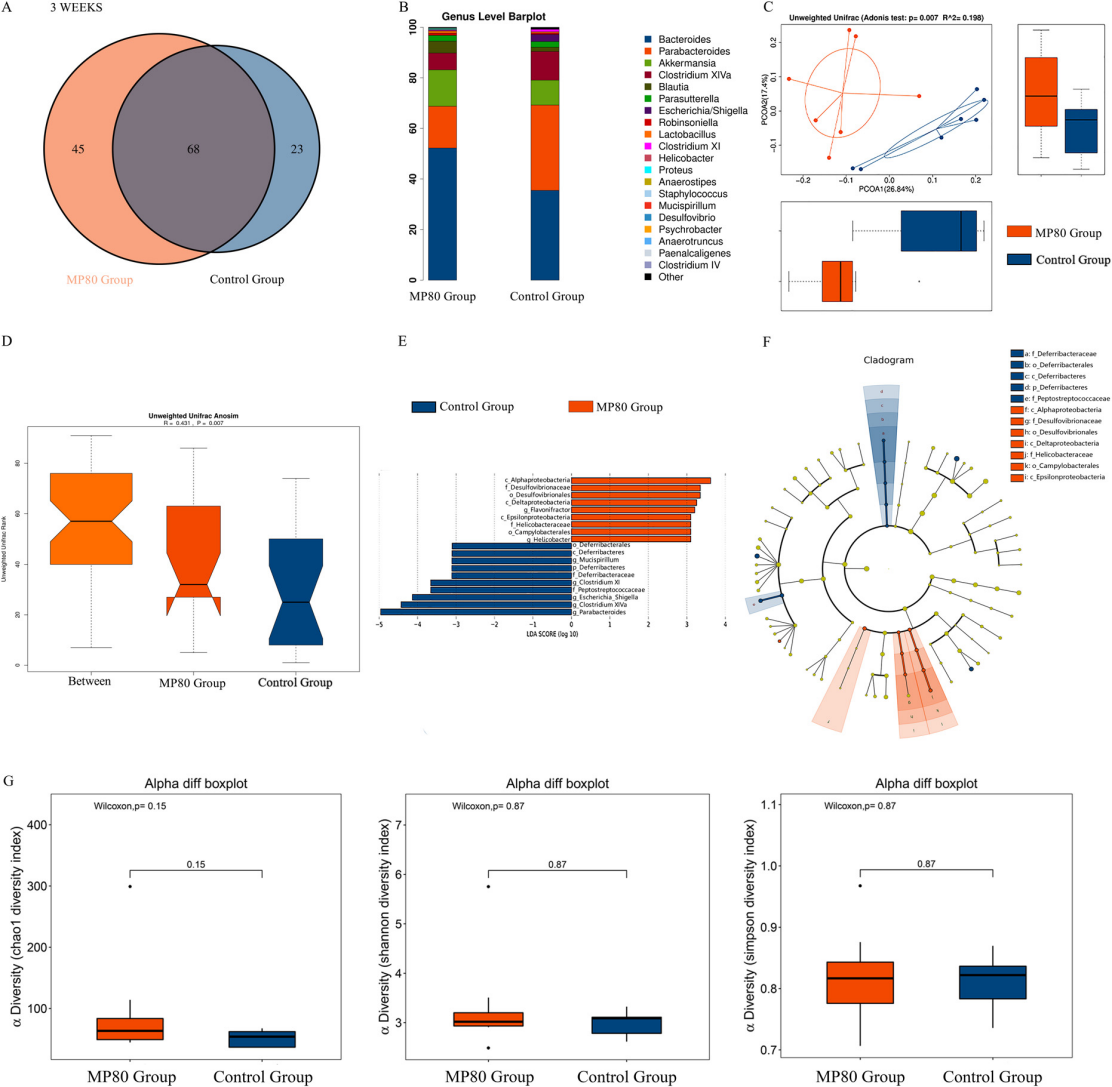
Maternal P80 intake-induced gut dysbiosis in 3-week-old offspring mice. (A) Venn diagram of OTUs. (B) Relative abundance of bacterial taxa at the genus level. (C and D) Unweighted UniFrac distance analysis (C) and unweighted UniFrac ANOSIM (D) between the two groups. (E and F) Differentially expressed microbiota (E) and differential levels in the cladogram (F) by LEfSe analysis. (G) α diversity was measured by chao1, Shannon diversity index, and Simpson diversity index. MP80, maternal P80. *n* = 7 in each group.

A Venn diagram was used to evaluate the operational taxonomic units (OTUs) in 3-week-old offspring of the two groups. Both groups shared 68 OTUs: 45 OTUs were solely found in the MP80 group, and 23 OTUs were solely found in the control group (Fig. 5A). The microbiota community structures of the two groups were remarkably different. Five dominant genera, namely, *Bacteroides*, *Parabacteroides*, *Akkermansia*, *Clostridium XIVa*, and *Blautia*, were found in various quantities in the MP80 group and control group (Fig. 5B). *Bacteroides*, *Akkermansia*, and *Blautia* were increased, whereas *Parabacteroides* and *Clostridium XIVa* were decreased, in the MP80 group compared with the control group (see Fig. S1A in the supplemental material). Principal-coordinate analysis (PCoA) on unweighted UniFrac distances was applied to characterize the β diversity of the two groups. PCoA analysis (Fig. 5C) and analysis of similarity (ANOSIM) (Fig. 5D) exhibited substantial differences between the two groups. We then studied the abundance of gut microbiota in the two groups. We found that Deltaproteobacteria and *Epsilonproteobacteria* were increased in the feces of offspring in the MP80 group at 3 weeks in the class level. The Deltaproteobacteria include *Desulfovibrionales* and *Desulfovibrionaceae*, and the *Epsilonproteobacteria* include *Campylobacterales* and *Helicobacteraceae* (Fig. 5E and F). No difference in α diversity was found between the two groups (Fig. 5G).

10.1128/mSystems.01337-20.1 FIG S1 The abundance of dominant genera after maternal P80 intake. Shown are the differences in the five dominant genera between the MP80 (maternal P80) group and control group in 3-week-old (A) and 8-week-old (B) mice. *n* = 7 in the 3-week-old group, and *n* = 5 in the 8-week-old group. *, *P* < 0.05; **, *P* < 0.01; ***, *P* < 0.001. Download FIG S1, TIF file, 0.3 MB. Copyright © 2021 Jin et al. 2021 Jin et al. https://creativecommons.org/licenses/by/4.0/ This content is distributed under the terms of the Creative Commons Attribution 4.0 International license.

**FIG 6:**
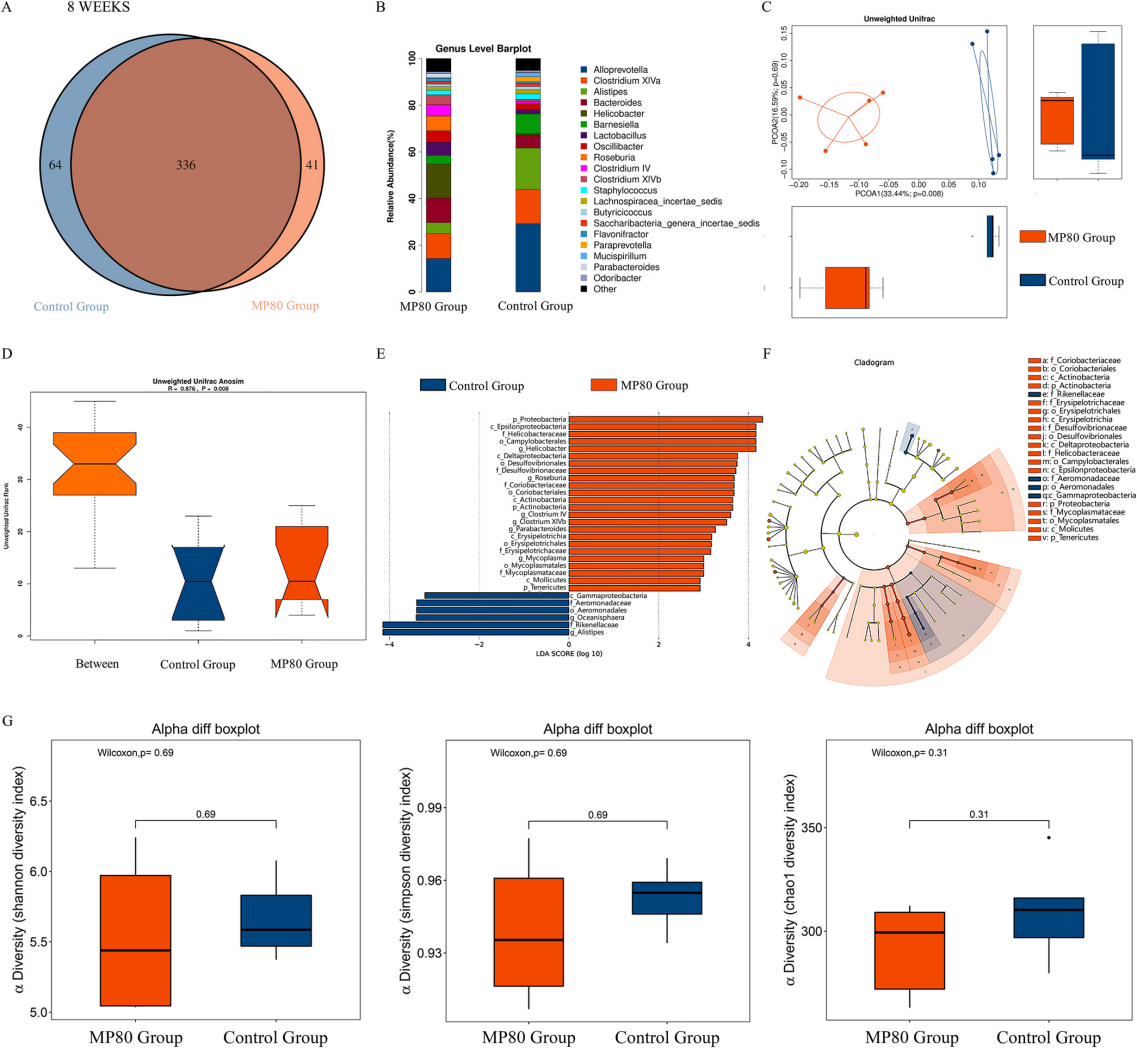
Maternal P80 intake altered the composition of gut microbiota in 8-week-old offspring mice. The composition and diversity of gut microbiota in 8-week-old offspring mice between the two groups remained different. (A and B) Venn diagram (A) and relative abundance (B) of bacterial taxa at the genus level. β diversity in the two groups was measured by (C) unweighted UniFrac distance analysis and (D) unweighted UniFrac ANOSIM. (E and F) Differentially expressed microbiota (E) and differential levels in cladogram (F) by LEfSe analysis. (G) α diversity was measured by chao1, Shannon diversity index, and Simpson diversity index. MP80, maternal P80. *n* = 5 in each group.

Surprisingly, the gut microbial compositions in 8-week-old offspring in the two groups still differed even without P80 treatment after weaning. The Venn diagram in Fig. 6A shows that shared OTUs were shared, 41 OTUs were found in the MP80 group exclusively, and 64 OTUs were found in the control group exclusively. The relative abundances of bacterial taxa at the genus level were different. Five dominant genera, namely, *Alloprevotella*, *Clostridium XIVa*, *Alistipes*, *Bacteroides*, and *Helicobacter*, were different between the MP80 group and control group (Fig. 6B). *Bacteroides* and *Helicobacter* were increased, whereas *Alloprevotella*, *Clostridium XIVa*, and *Alistipes* were decreased in the MP80 group (Fig. S1B). PCoA analysis and ANOSIM showed a remarkable difference in the β diversity between the two groups (Fig. 6C and D). Some microbiota that contain flagella, such as *Proteobacteria*, *Desulfovibrionales*, *Epsilonproteobacteria*, and *Helicobacteraceae*, which play a critical role in IBD, were higher in the MP80 group than the control group. *Alistipes*, *Rikenellaceae*, and *Oceanisphaera* were higher in the control group than the MP80 group (Fig. 6E and F). No difference in α diversity was found between the two groups in 8-week-old mice (Fig. 6G). *Alistipes* and *Rikenellaceae* can maintain normal colon function and play a crucial part in the shape and function of colonic epithelial cells (44, 45).

**FIG 7:**
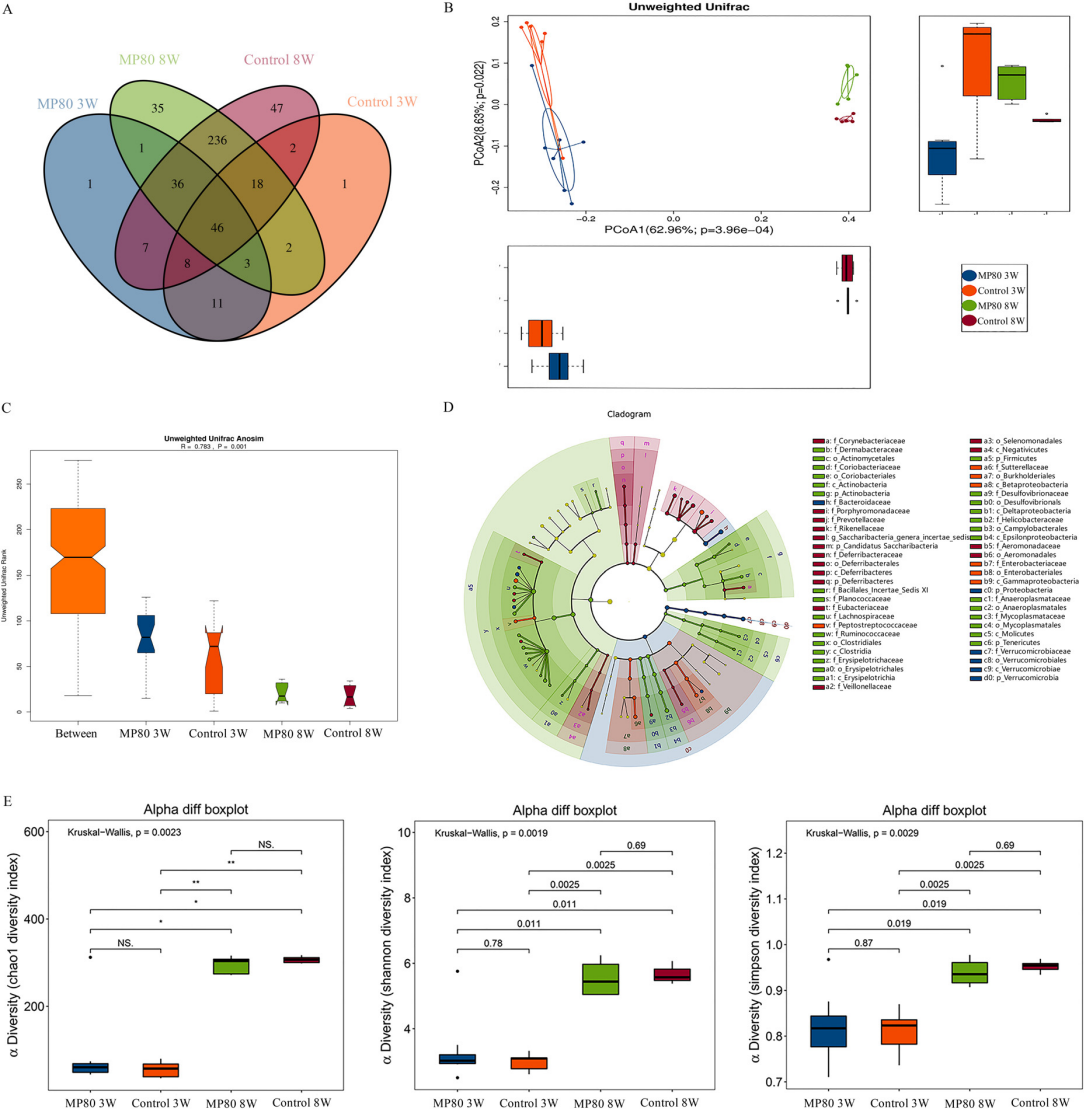
Maternal P80 intake-induced dysbiosis persists from the 3rd week to the 8th week of offspring. Shown are the gut microbiota in 3- and 8-week-old offspring. (A) The Venn diagram showed an overlap of four groups. (B) Characteristics of infant and adult gut microbiota as illustrated by PCoA clustering analyses. (C) ANOSIM results showed a substantial alteration of the gut microbiota between the two groups. (D) Known genera reported by LEfSe in the comparison of the bacterial community in 3- and 8-week-old mice in the MP80 group and control group. (E) α diversity was measured by chao1, Shannon diversity index, and Simpson diversity index. MP80, maternal P80.

We jointly analyzed the microbiota evolution of the two groups of mice at 3 and 8 weeks by using 16S rRNA sequencing. The Venn diagram in Fig. 7A shows an overlap between the MP80 group and control group. PCoA at 3 and 8 weeks of age showed that no longer giving P80 after weaning could partially reverse the effect of P80 on the microbiota of the offspring (Fig. 7B). Unweighted Unifrac ANOSIM analysis revealed that the differences among the four groups were substantial (Fig. 7C). Inflammation-associated microbiota, including *Actinobacteria*, *Erysipelotrichia*, and *Proteobacteria*, were increased. *Proteobacteria*, *Desulfovibrionaceae*, and *Helicobacteraceae* have the same trend in 3- and 8-week-old offspring (Fig. 7D). The difference in α diversity was found between the 3-week-old and 8-week-old mice in the two groups (Fig. 7E).

### Predictive function analysis.

**FIG 8:**
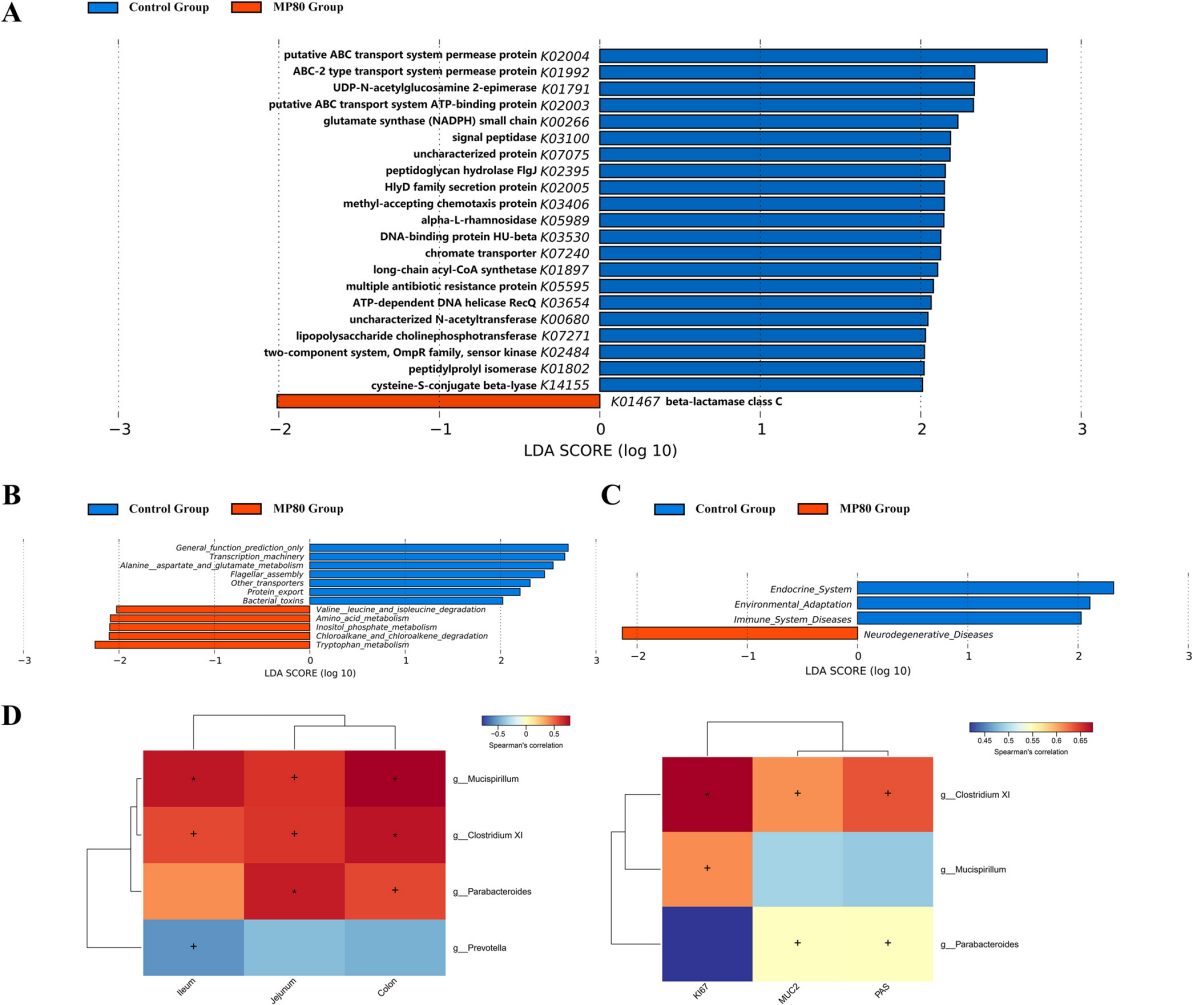
Functional predictions for the intestinal microbiome of 3-week-old offspring after maternal P80 intake. (A) The KOs with significantly different abundances in the intestinal microbiota are shown. Significant KEGG pathways at level 2 (B) and level 3 (C) for the intestinal microbiota of the control group and MP80 group. (D) Heatmaps showing correlations between microbiota genera and intestinal length and positive cells by Ki67, MUC2, and PAS. KEGG, Kyoto Encyclopedia of Genes and Genomes; KO, KEGG orthologs; MP80, maternal P80. +, *P* < 0.05, and *, *P* < 0.01, by Spearman’s test.

PICRUSt based on closed-reference OTU was used to predict the abundances of functional categories of the KEGG ortholog (KO). A total of 22 KOs were identified with significantly different abundances in the fecal microbiome between the MP80 group and control group (false-discovery rate [FDR], *P* < 0.05) (Fig. 8A). In the level 2 and 3 KEGG pathways, the microbial gene functions, including those associated with neurodegenerative diseases, amino acid metabolism, and inositol phosphate metabolism, were increased in MP80 group, while those associated with the endocrine system, environmental adaptation, and protein export were decreased (Fig. 8B and C).

### Association between intestinal microbiota and proliferation and differentiation.

We found the correlations between intestinal microbiota and proliferation/differentiation, particularly intestinal length, and positive cells in Ki67, MUC2, and PAS. The fecal microbiota (*Mucispirillum*, *Clostridium XI*, and *Parabacteroides*; *P* < 0.05) were positive correlated with intestinal length, while *Prevotella* was negatively correlated with ileum length (*P* < 0.05). Interestingly, we also found that *Clostridium XI* was positively associative with Ki67, MUC2, and PAS (*P* < 0.05), *Mucispirillum* with Ki67 (*P* < 0.05), and *Parabacteroides* with MUC2 and PAS (*P* < 0.05) (Fig. 8D).

### Maternal P80 intake promoted DSS-induced colitis in offspring in adulthood.

**FIG 9:**
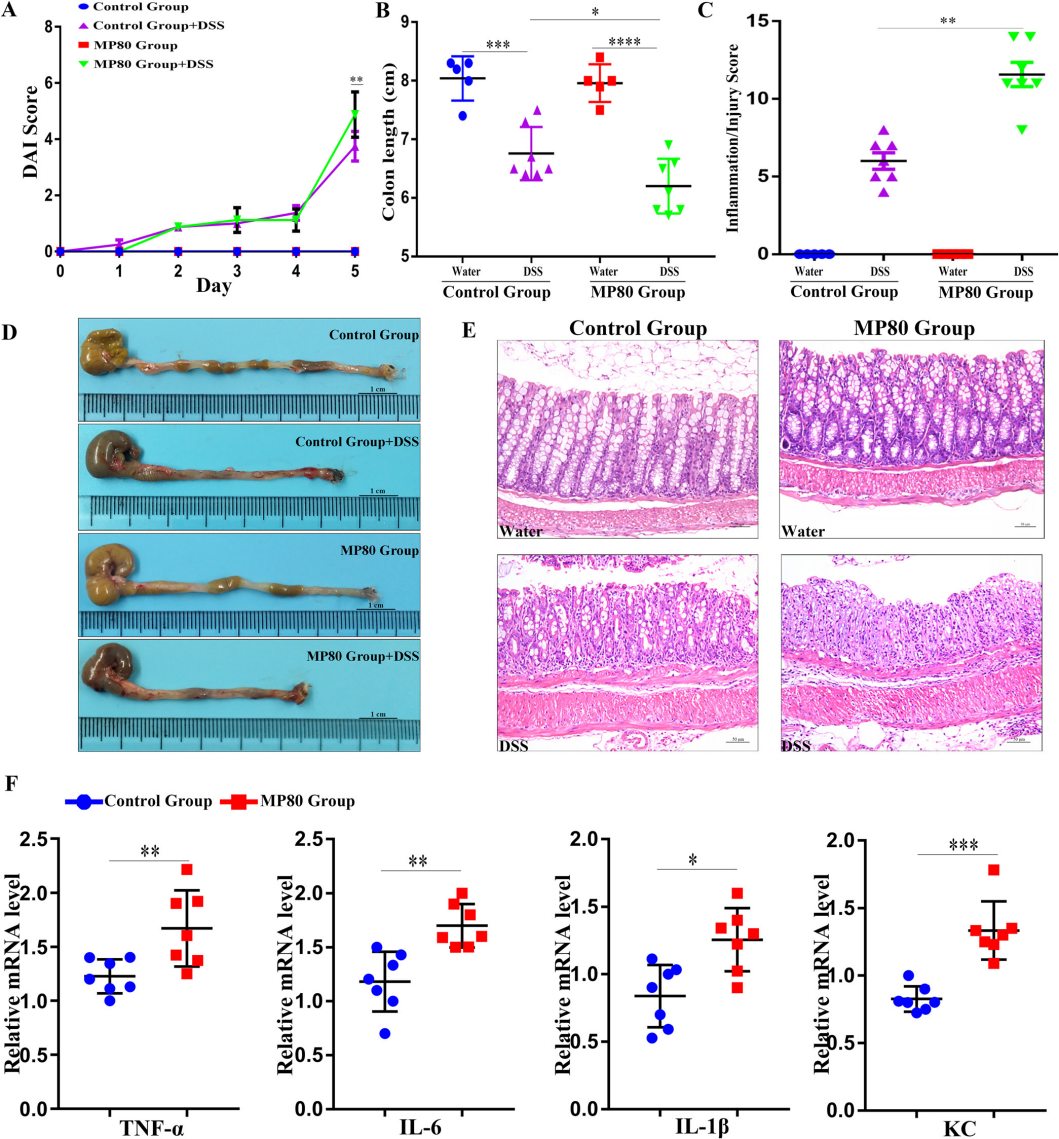
Maternal P80 intake exacerbated DSS-induced colitis of offspring in adulthood. (A) Daily DAI. (B) Colon length. (C) Inflammation/injury score. (D) The lengths of the colon and ileocecal portions of mice. (E) Paraffin-embedded colon sections stained with H&E. (F) The relative expression of TNF-α, IL-6, IL-1β, and KC is presented. MP80, maternal P80. Scale bars, 50 μm. *n* = 5 each in the MP80, control, MP80 DSS, and control DSS groups. *P* values of >0.05 are not significant. *, *P* < 0.05; **, *P* < 0.01; ***, *P* < 0.001; ****, *P* < 0.0001.

The immune effects of microbial exposure in early life can persist into the later stage of life and are associated with IBD susceptibility. In the present study, DSS treatment induced more severe colitis in the MP80 group than the control group (5.571 ± 1.272 versus 3.750 ± 1.488; *P* < 0.01) (Fig. 9A). Colonic tissues shrank after the DSS challenge because of inflammation, and a substantial shortening of the colon was observed in the MP80 group (Fig. 9B). Additionally, the inflammatory/injury score in the MP80 group (11.000 ± 2.507) was significantly higher than that in the control group (6.750 ± 2.493; *P* < 0.01) (Fig. 9C). The colon and ileocecal portions of the mice were removed to measure their lengths (Fig. 9D). Inflammatory histological changes emerged because of the increase in neutrophil and lymphocyte infiltration and the extensive injury of intact crypt structures and surface epithelium (Fig. 9E). The relative mRNA expression of TNF-α, IL-6, IL-1β, and chemokine (C-X-C motif) ligand 1 (KC) was elevated in the MP80 group compared with the control group (Fig. 9F). Moreover, the exacerbation of colitis in the MP80 group was confirmed by H&E staining. Together, these data suggest that maternal P80 intake substantially aggravated the offspring’s susceptibility to colitis in adulthood.

## DISCUSSION

Several environmental factors, including diet (46), medication (47), and modes of delivery (48), may be correlated with the occurrence of IBD. However, emulsifiers, as common food additives, are sometimes neglected. Although emulsifiers might induce low-grade inflammation (5), the specific correlation between maternal P80 exposure and IBD has not been reported.

The global food emulsifier market is a promising market segment in the food industry. The food emulsifier market is considered the fastest-growing part of the food additive market because the decline in fat content in food is becoming increasingly obvious. According to published reports, the food emulsifier market grew by 35% to over a 2.8-million-dollar industry from 2012 to 2018. The global food emulsifier market is expected to reach 933,400 tons by 2018 because of the increasing demand for emulsifiers (49).

Emulsifiers are a class of compounds that make a mixed liquid of two or more incompatible components (50) and are ubiquitously applied in the manufacture and processing of food, pharmaceuticals, and cosmetics (51). Epidemiological studies found that the incidence of autoimmune diseases has risen concerning the disruption of intestinal tightness with the increased application of industrial food additives, especially emulsifiers (49). Reducing the intake of processed foods and emulsifiers may relieve intestinal inflammation (52). E. Viennois found that P80 supplementation in mice could cause the destruction of the intestinal barrier and the reduction of the mucus layer, increase inflammation, reduce *Bacteroidales*, and increase Ruminococcus gnavus (53). B. Chassaing et al. demonstrated that mice treated with 1% P80 dissolved in drinking water for 12 weeks had low-grade inflammation, obesity/metabolic syndrome, and colitis (5). Moreover, H. Furuhashi et al. also found 1% P80 could induce small intestine vulnerability to indomethacin-induced lesions (54). Thus, we also chose 1% P80 as the intervention concentration. Our study focused on whether maternal P80 intake induces gut dysbiosis in offspring and increases the susceptibility to colitis in adulthood. We found a remarkable difference in the offspring after 6 weeks of maternal P80 intervention.

Emulsifier supplementation could lead to weight gain (55). In our study, offspring in both groups had no remarkable difference in body weights. Hence, we speculated that the maternal supplementation of emulsifiers caused long-term negative effects in the offspring that may be independent of obesity. Chassaing et al. reported that the supplementation of emulsifiers would damage the inner mucus layer, and bacteria would more easily invade intestinal cells and impair the integrity of the intestinal epithelium (5). We found that the intestinal barrier of the offspring was impaired, which resulted in low-grade intestinal inflammation and dysbiosis. In our results, the expression of MUC2, tight junction protein, and sIgA (56) in the intestinal tissues of the offspring in the MP80 group were reduced.

The intestinal mucosal barrier can maintain the ability to absorb nutrients while providing enough lumen for microorganisms and molecules. The secretion of mucus-forming mucus protein, sIgA, and antimicrobial peptide strengthens the outer mucosal barrier, while various immune cells participate in mucosal defense in the inner layer (56). These acts form the defense function of the intestinal barrier and prevent the invasion of pathogens and the activation of the immune response. Mucus layer defection enhances the susceptibility of the pathogen (57). The epithelial paracellular barrier is composed of intercellular contacts called tight junctions (TJs). The TJ plays an important role in maintaining the integrity of the intestinal barrier. Studies have shown that mice with colon claudin-7 deficiency develop fatal colitis shortly after birth (58). sIgA secreted by epithelial cells also maintains the integrity of the intestinal barrier and prevents bacterial invasion. The loss of barrier integrity increases the translocation of bacterial antigens and stimulates the inflammatory response of intestinal mucosa. We speculate that the excessive degradation of mucin may disrupt the integrity of the mucosal barrier and promote the colonization of intestinal pathogens, which cause inflammation.

How did the offspring present an impaired intestinal barrier despite having no direct access to P80? One hypothesis is that P80 can be delivered from mothers to their offspring through the placenta or breast milk. The absorption of P80 in the intestine is minimal: 91% of P80 is excreted in the feces, and 2.1% is excreted in the urine (59). A tiny amount is degraded in the intestines (60). The emulsifier is composed of a hydrophilic end and a lipophilic end. The lipophilic end is absorbed through standard fat absorption mechanisms. The hydrophilic moieties will be defecated in an inert form without being absorbed or metabolized. Hence, P80 cannot be delivered through the placenta or breast milk in an active form (3). M. K. Holder found that emulsifiers might affect anxiety-like and social-related behaviors in female mice after 15 weeks of treatment. However, no obvious changes in behaviors occurred in the 12th week of treatment. Therefore, we believe that maternal behaviors may not have a considerable effect on the growth and development of offspring in the 9th week in our experiment (61).

Another explanation is that P80-induced gut dysbiosis was transmitted from mothers to their offspring and somehow perturbed the proper formation of the intestinal barrier and the development of the gut. Studies have confirmed the existence of microbiota transmission from mother to infant through feces, the birth canal, breast milk, and skin (21–26). P80 could independently alter microbiota composition according to an *ex vivo* M-SHIME model, which could mimic a complex and stable human microbiome without a live host (62). Our study also revealed that gut dysbiosis occurred in the offspring at weaning and adulthood. Therefore, the long-term negative effects of emulsifiers on the host are more likely to be caused by the transmission of the microbiota.

Studies have suggested that mother-to-infant microbiome transmission routes are necessary in the development of the infant microbiome (63). The transmission of mother-to-child microbiota plays a critical role in the establishment and development of infant intestinal microbiota. The mother’s gut microbiome was found to be the largest donor of infant-acquired strains and was easier to colonize (64). Interestingly, P. Ferretti et al. collected 25 pairs of bacterial microbiotas from different locations of mother and infant. Analysis of species and strain levels showed that microorganisms from mothers were continuously transmitted to infants, and the strains passed from mothers to infants mainly came from the intestinal microbiota (25).

Chassaing et al. have reported that P80 can impact the mouse gut microbiota promoting colitis (5). Therefore, we compared the gut microbiota in the offspring of this experiment with that of Chassaing’s study. Surprisingly, we found that there was consistency in the changes of the key microbiota. *Lachnospiraceae*, *Ruminococcaceae*, *Coriobacteriaceae*, and *Helicobacteraceae* were the main changed microbiota in the offspring of 3 and 8 weeks in our study, as well as those in Chassaing’s study.

We performed fecal microbiota transplantation (FMT) to confirm this inference. Interestingly, the results showed that the feces of MP80 offspring could aggravate the intestinal inflammation and disrupt the intestinal barrier. Therefore, our data supported that P80 exposure in early life disrupts the offspring's intestinal barrier in adulthood by inducing gut microbiota dysbiosis.

Our results and studies showed that intestinal microbiota structure in early life has a profound impact on infant immune development, and maternal factors play a key role in the intestinal microbiota of offspring (65). Maternal microbes could enter the gastrointestinal tract of the fetus, and the delivery and feeding modes are important for gut microbiota (65, 66). Previous studies have shown that P80 intake could change intestinal microbiota and increase intestinal permeability in mice (5). Therefore, we speculated whether the negative effects of P80 exposure can be passed on from mother to offspring during pregnancy and lactation, which are critical to the formation of infant gut microbiota. Our results supported that this negative effect is transmitted between mother and infant. The interaction between the two successive periods needs to be completely cut off to determine which stage is more important. To eliminate the interference of lactation factors, different treatment groups of newborn offspring need to be separated from their mothers and fostered by another group of female mice. As for determining the role of lactation, the newborn offspring of untreated female mice should be fostered by lactating female mice who have either been exposed to P80 or not. Due to technical limitations, we are unable to achieve it at present.

The gut microbiota system, which contains trillions of bacteria, is affected by many factors. Changes in intestinal microbiota may be a result of changes in the intestinal environment. We confirmed MP80 induced-gut dysbiosis in the offspring when they were still infants, and this alteration in microbial composition was sustained until 8 weeks postpartum, even though these P80-treated maternal offspring took in sterilized water and chow and did not have direct access to P80. This finding is consistent with the well-recognized fact that the development of the infant’s gut microbiota is accomplished in the early stage of life (16). In the present study, *Proteobacteria*, *Helicobacteraceae*, *Campylobacterales*, and *Desulfovibrionales* were present in the flora at 3 and 8 weeks; hence, these bacteria continue to play an essential role in intestine inflammation, which leads to susceptibility to colitis in adulthood. *Proteobacteria* can cause inflammation, alter intestinal flora, and therefore promote malnutrition and the development of IBD (67).

**FIG 10:**
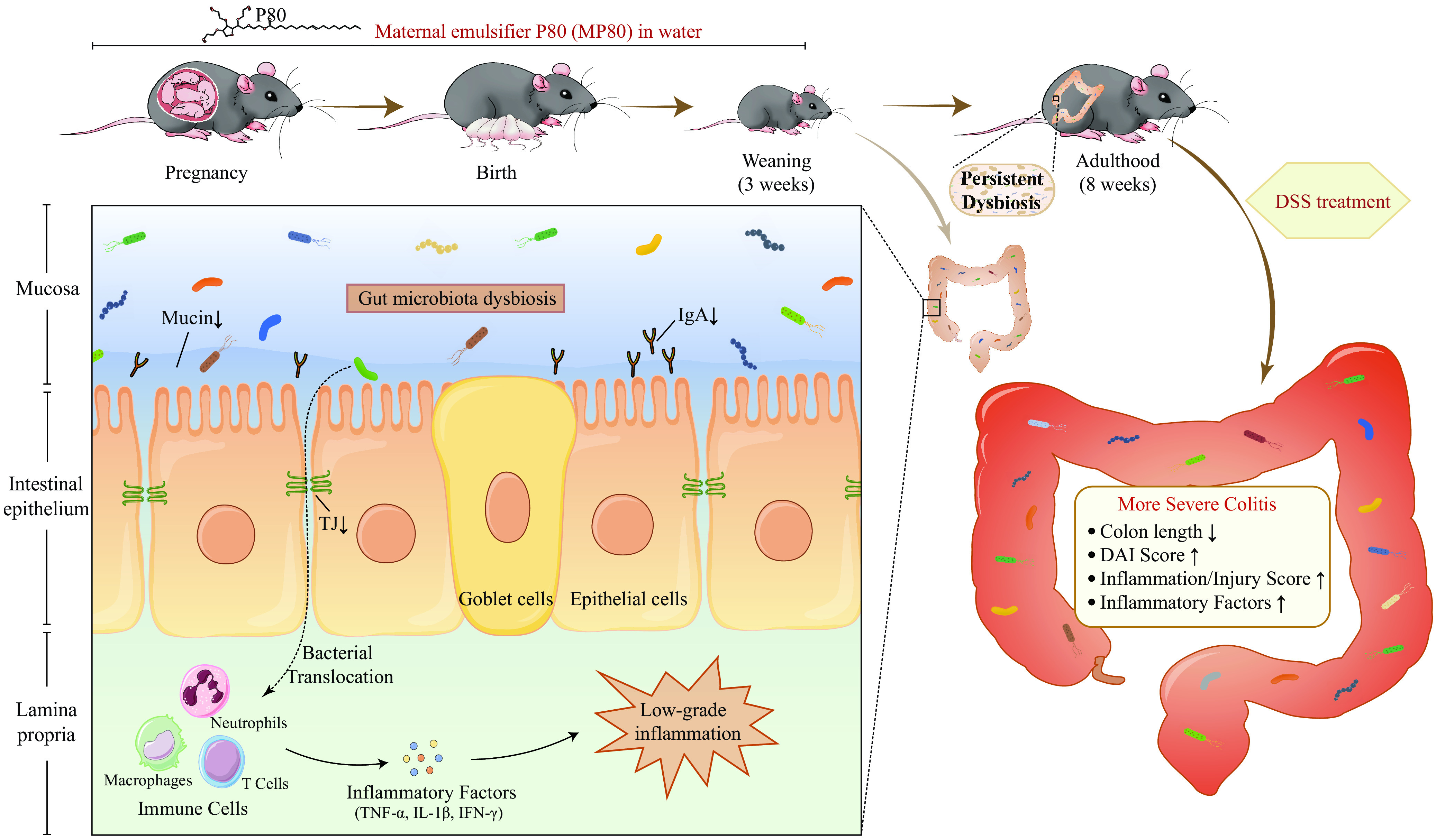
Maternal emulsifier P80 intake induces gut dysbiosis in offspring and increases the susceptibility of colitis in adulthood. The intake of P80 during pregnancy and lactation resulted in dysbiosis of intestinal microbiota, impairment of mucus and immune barrier function, and reduction of the intestinal epithelial tight junction in offspring. With the disruption of the intestinal barrier, bacterial translocation increases and stimulates the immune response, leading to the occurrence of low-grade inflammation. This negative effect continued into adulthood and aggravated colitis after DSS modeling. The symptoms were shorter colon length and higher DAI, inflammatory/injury score, and inflammatory factors. DAI, disease activity index; DSS, dextran sulfate sodium; TJ, tight junctions; IgA, immunoglobulin A; TNF-α, tumor necrosis factor-α; IL-1β, interleukin 1β; IFN-γ, interferon gamma.

J. Mirpuri et al. (68) reported that *Proteobacteria*, which represent immature flora, are persistent in mice that lack sIgA. Proteobacterial colonization in early life can trigger a persistent inflammatory status. Supplemental *Helicobacteraceae* can cause high production of IL-12, which leads to a T helper type 1 (Th1)-polarized response and elevated levels of Th1 cytokines (69). The *Epsilonproteobacteria*, which are an important food pathogen, contain flagella, which may activate TLR5 targets in early life and cause susceptibility to adult colitis. Besides, *Campylobacterales* is positively associated with colitis. The release of degrading enzymes and oxidation products produced by Campylobacter jejuni neutrophils can cause extensive collateral tissue damage to the host and cause colitis (70). *Desulfovibrionales* can degrade sulfated mucin in the intestinal mucosa and colonize the intestine through the mucus layer of the intestine (70, 71). Excessive growth of *Desulfovibrionales* in the intestine can aggravate the intestinal flora imbalance, affect the symbiotic relationship between the intestinal flora and the host, promote IL-6 secretion, and induce an inflammatory response (72). Moreover, the metabolite H_2_S of *Desulfovibrionales* can penetrate cell membranes freely and block the butyrate oxidation pathway of intestinal epithelial cells, and it has a toxic effect on cells (73). 16S rRNA sequencing and correlation analysis revealed that *Mucispirillum*, *Clostridium XI*, and *Parabacteroides*, which positively correlated with intestinal proliferation and differentiation, were decreased in the MP80 group. Dysbiosis and intestinal barrier dysfunction are regarded as two crucial pathogenic factors involved in triggering IBD (12–14, 74, 75). Our study also demonstrated that the offspring of P80-treated mothers were more vulnerable to DSS-induced colitis. This finding offers an insight into the potential pathogenic property of emulsifiers in early life, although we still have not clarified the exact mechanisms by which dysbiosis in early life induced by MP80 interferes with the gut of offspring in the current study (Fig. 10). Further studies are on the way.

## MATERIALS AND METHODS

### Animals and treatment.

The F_0_ generation consists of 10 male and 10 female C57BL/6 mice (6 weeks old) purchased from Beijing HFK Bioscience Co., Ltd., China, and housed five per cage. All mice were fed in the animal center of Tianjin Medical University under specific-pathogen-free conditions with air filtration (22 ± 2°C) in a 12-h/12-h light/dark cycle and free access standard rodent chow (calories at 3.76 kcal/g, 16.7 E% fat, 19.3 E% protein, 64 E% carbohydrates [AIN-93G; Research Diets, Guangdong, China]) and sterilized water. The F_0_ male mice were supplied with sterilized water without P80. The F_0_ female mice were randomly divided into two groups: the P80 group and the control water group. The same group of mice was housed in one litter. Mice from the P80 group were exposed to P80 dissolved in sterilized water (1.0% [wt/vol]), whereas the control water group was supplied with sterilized water without P80. The dose was chosen based on a previous study (5, 54). Female mice in the two groups were mated with male mice after 3 weeks of predisposing with sterilized water or P80 dissolved in sterilized water and checked daily. Pregnant mice within the same group were housed together up until 1 week before the estimated parturition moment. Then, they were housed solely in separate cages until delivery.

Subsequently, the offspring were delivered vaginally, and their body weight was monitored at birth and weekly for 8 weeks. The offspring of the P80 group were named the MP80 group, and those of the control water group comprised the control group. We obtained 38 pups: 19 in the MP80 group and 19 in the control group. The offspring were weaned at around 3 weeks of age, separated from the mothers, and housed with five mice per cage. In the third week, seven mice each were randomly selected from each group to be sacrificed. The remaining offspring were caged by sex in the same group and housed with five mice per cage. All offspring received sterilized drinking water without P80 until the 8th week after separation. In the eighth week, five offspring each were randomly selected from each group to be sacrificed. The remaining offspring were treated with DSS for 5 days before being sacrificed.

### FMT.

We collected 600 mg fresh feces from 3-week-old offspring from both groups and placed the feces in 15 ml of sterile phosphate-buffered saline (PBS) solution with 0.05% cysteine hydrochloride. In an anaerobic environment, the fecal liquid was shaken for 3 min and stood for 2 min in ice. We used a Hungate anaerobic tube to collect the supernatant. The supernatant was stored in a refrigerator (−80°C). Ten 8-week-old C57BL/6 mice were randomly divided into two groups: the FMT-P group (gavaged with fecal samples from the MP80 group) and the FMT-C group (gavaged with fecal samples from the control group). All mice received antibiotic cocktails mixed with 200 mg/liter ampicillin, metronidazole, neomycin, and 100 mg/liter of vancomycin in drinking water for 5 days according to the previous studies (76). The fecal supernatant from two groups was gavaged three times per week for 1 month. The amount of gavage was 200 μl each time per mice.

### Tissue collection.

The 3-week-old and 8-week-old offspring mice were separately placed in a sterilized empty cage for 1 h, and fecal samples were collected into a sterile tube (approximately 150 mg of fresh feces) 2 days before the mice were sacrificed. Feces were stored in an −80°C refrigerator. The 3-week-old and 8-week-old offspring mice were sacrificed under anesthesia. Intestines from the mice were removed and flushed with cold PBS until no luminal content was evident. The intestines were cut into four pieces (proximal, middle, and distal small intestines and colorectal sections) and opened longitudinally. The proximal part of each intestine segment was flash-frozen in liquid nitrogen and subsequently stored at −80°C. The distal part was rolled up and preserved in 4% paraformaldehyde solution.

### Histology and immunohistochemistry.

The intestinal tissues were fixed in 4% paraformaldehyde, dehydrated, embedded in paraffin, and cut into 4-μm-thick sections according to the standard H&E method. After deparaffinization and hydration, H&E staining was performed for the evaluation of intestinal development and inflammation. We measured the length of villi for small intestinal tissue and crypt depth for colonic tissue. Villus length is the vertical distance from the top of the villus to the opening of the crypt, and crypt depth is the vertical distance from the crypt opening to the crypt base (77–79). We randomly selected 100 well-orientated villi/crypts at a 200× magnification with a light microscope (Olympus, Japan).

For immunohistochemistry, tissue slices were incubated with primary antibodies, rabbit monoclonal anti-Ki67 (ab16667; Abcam), and anti-MUC2 (Santa Cruz Biotechnology) overnight at 4°C. Subsequently, the tissue sections were washed with PBS, incubated with biotinylated anti-rabbit or anti-goat secondary antibody (Santa Cruz Biotechnology) for 30 min at 37°C, and counterstained with horseradish peroxidase (HRP)-conjugated streptavidin solution. Target staining was determined by counting the absolute number of positively stained cells. One hundred villi or crypts were randomly selected to evaluate the positive rate of each section. The positive ratio of each group was determined by counting the average ratio of all sections within the group with a light microscope (Leica, Germany).

### PAS staining.

Paraffin-embedded colonic tissues were deparaffinized. The processed sections were incubated in 1% periodic acid solution (Sigma-Aldrich) for 10 min and stained with Schiff reagent (Sigma-Aldrich) for 40 min. Subsequently, the stained sections were counterstained with hematoxylin solution for 2 to 5 min. All steps were performed at room temperature, and the stained sections were rinsed with PBS solution after each step. Five vision fields were randomly selected in each section, and the number of goblet cells in each field was counted. The average number of goblet cells in each colonic gland was calculated.

### Immunofluorescent staining.

Paraffin-embedded tissues (4 μm thick) were deparaffinized, hydrated, and incubated for 15 min for antigen retrieval. A blocking buffer was used to block nonspecific binding. The primary antibodies used were an anti-IgA (rabbit anti-mouse; Abcam) and anti-ZO-1 antibodies (rabbit anti-mouse; Cell Signaling Technology), which were incubated overnight with the tissues at 4°C. Then, the sections were incubated with fluorochrome-conjugated secondary antibody IgG H&L (Cell Signaling Technology) at room temperature for 1 h. DAPI (4′,6-diamidino-2-phenylindole) was utilized to dye the nucleus. The processed sections were washed with PBS solution after each step. IgA- and ZO-1-positive cells were analyzed using a fluorescence microscope (DM5000 B; Leica, Germany). One hundred villi/crypts were randomly selected and viewed in each group.

### RT-PCR analysis.

We extracted the total RNA from the colon tissues by using TRIzol (Invitrogen, La Jolla, CA). Lithium chloride purification is a necessary step for the colon sample processing of DSS-treated mice. Total RNA was purified by lithium chloride precipitation. RNAs were dissolved in 270 μl RNase-free water and added to 30 μl of 8 M LiCl solution. All solutions were thoroughly mixed, incubated in ice for 2 h, and centrifuged at 14,000 × *g* for 30 min at 4°C. The supernatant was discarded, and the pellet was dissolved in 200 μl RNase-free water. We repeated this step once, and the pellet was dissolved in 90 μl RNase-free water in the last step. RNA solution was mixed with 10 μl of 3 M sodium acetate and 200 μl anhydrous ethanol and incubated at −20°C for 30 min. All solutions were centrifuged at 14,000 × *g* for 30 min at 4°C to obtain a pellet. The pellet was washed with 50 μl of −20°C prechilled 75% ethanol and centrifuged at 14,000 × *g* for 10 min at 4°C. Then, the RNA was subjected to cDNA reverse transcription (RT) according to the manufacturer’s instructions of the TIAN Script RT kit (Tiangen, Inc., Beijing, China). RT-PCR analysis was performed using TaqMan Gene Expression Master Mix and primers (Genewiz, Inc., Beijing, China). The oligonucleotide primers for target genes, including those coding for TNF-α, IFN-γ, IL-1β, IL-6, KC, ZO-1, OCLND, CLND-3, MUC2, APRIL, and glyceraldehyde-3-phosphate dehydrogenase (GAPDH), are listed in Table S1 in the supplemental material. Each cDNA of the sample was analyzed in triplicate, and the expression was normalized to GAPDH. The 2^−ΔΔCT^ (threshold cycle) method was applied to calculate the relative mRNA expression of each target gene.

10.1128/mSystems.01337-20.2 TABLE S1 Oligonucleotide primers used in real-time PCR. All primer pairs and primer sequences are listed. “F” and “R” in the primer name represent forward and reverse primers, respectively. Download Table S1, DOCX file, 0.02 MB. Copyright © 2021 Jin et al. 2021 Jin et al. https://creativecommons.org/licenses/by/4.0/ This content is distributed under the terms of the Creative Commons Attribution 4.0 International license.

### Western blot.

The colonic tissues were immersed in radioimmunoprecipitation assay buffer with protease inhibitors (Solarbio, Beijing, China) and homogenized. Afterward, the protein concentration of homogenates was detected by bicinchoninic acid protein assay (Thermo Scientific, Inc.). The membranes were incubated overnight at 4°C with primary antibodies to CLND-3 (rabbit anti-mouse; Abcam) and ZO-1 (rabbit anti-mouse; Cell Signaling Technology) and then incubated with HRP-conjugated secondary antibodies (Cell Signaling Technology). Band intensity was quantified by ImageJ software.

### Gut microbiota analysis.

Fecal specimens of the 3-week-old and 8-week-old offspring were collected to extract the total fecal bacterial DNA with a QIAamp Fast DNA Stool minikit (QIAamp, Germany) according to the manufacturer’s introduction. The mice were placed in a sterile box, and a sterile tube was used to collect the feces while avoiding urine contamination. The feces were immediately stored in liquid nitrogen after collection. The 16S V3-V4 hypervariable region was amplified for sequencing using the forward (F) and reverse (R) fusion primers 341F (CCTAYGGGRBGCASCAG) and 806R (GGACTACNNGGGTATCTAAT). Fecal microbiota composition was detected by 16S rRNA sequencing (Realbio Genomics Institute, Shanghai, China) using an Illumina HiSeq platform (Illumina, San Diego, CA, USA).

Barcode sequence and PCR amplification primer sequence were used to split the data of each sample from the original data of the deplane. The barcode and primer sequences were truncated, and the readable area of each sample was spliced by FLASH. The splicing sequences obtained were the raw tags. The raw tags were filtered to produce clean tags. We refer to QIIME's tag quality control process to capture tags and filter the length of tags. The interception of tags was performed to truncate the original tags from the first low-mass base site where the base number reaches the set length (a default mass threshold of ≤19) from the continuous-low-mass value. Tag lengths were filtered by intercepting the tags and further filtering out the tags with a continuous high-quality base length of less than 75% of the tags. The obtained tags were processed to remove the chimeric sequence to obtain the final effective data. USEARCH (V7.0.1090) was used to cluster the labels with 97% OTU similarity after dropping the singleton and removing the chimera. The Vegan package in the R language tool was used for the analysis of community heat maps, and VEGDIST and Hclust were used for distance calculation and cluster analysis, respectively. The distance algorithm used was Bray-Curtis, and the clustering method was Complete. Investigation of Communities by Reconstruction of Unobserved States (PICRUSt) was used to predict the abundances of functional categories in the Kyoto Encyclopedia of Genes and Genomes (KEGG) orthologs (KOs).

The resulting matrix of distances was performed by principal-component analysis of the two groups. Abundance and diversity were estimated by Shannon and Simpson indexes. The heat map was created using R. Cluster analysis was used to compare the differences between groups. The clustering tree was used to analyze the distribution of different bacteria. Different colors represent different groups, and nodes of different colors represent the microbial groups that play an important role in the group represented by the color. Yellow nodes indicate the groups of microorganisms that did not play an important role in different groups. The linear discriminant analysis threshold was 2.

### DSS-induced colitis.

Colitis was elicited in 8-week-old mice offspring using 2% DSS (36 to 50 kDa; MP Biomedicals) dissolved in sterilized water for 5 days. The DSS solution was changed daily until the end of the study. Colitis severity was assessed using the disease activity index (DAI) based on weight loss, the characteristics of feces, and the extent of hematochezia. The mice were sacrificed, and the entire colon was immediately excised for the measurement of colon length. The histological injury was quantified via a scoring system based on inflammation severity (scale of 0 to 3), the ratio of inflammatory fields (0 to 4), crypt impairment (0 to 4), and the depth of injury (0 to 3) by an experienced pathologist who was blind to the study (80).

### Statistical analysis.

SPSS 22.0 (SPSS, Chicago, IL, USA) was applied to all data analysis. Data are presented as means ± standard errors of the means, and the difference between groups was compared by Student’s unpaired *t* test. A *P* value of <0.05 was considered statistically significant. A one-way analysis of variance (ANOVA) was used for comparison between groups. *Post hoc* Tukey’s comparisons were used to test for pairwise differences. Tukey’s test was used to generate a critical value to control the familywise error rate. The statistical description of the count data was used for the composition ratio.
