# Supplementary figures and images for: Maternal Emulsifier P80 Intake Induces Gut Dysbiosis in Offspring and Increases Their Susceptibility to Colitis in Adulthood
Source: mSystems. 2021 Mar 16;6(2):e01337-20. doi: 10.1128/mSystems.01337-20 (PMC8547008; doi:10.1128/mSystems.01337-20)

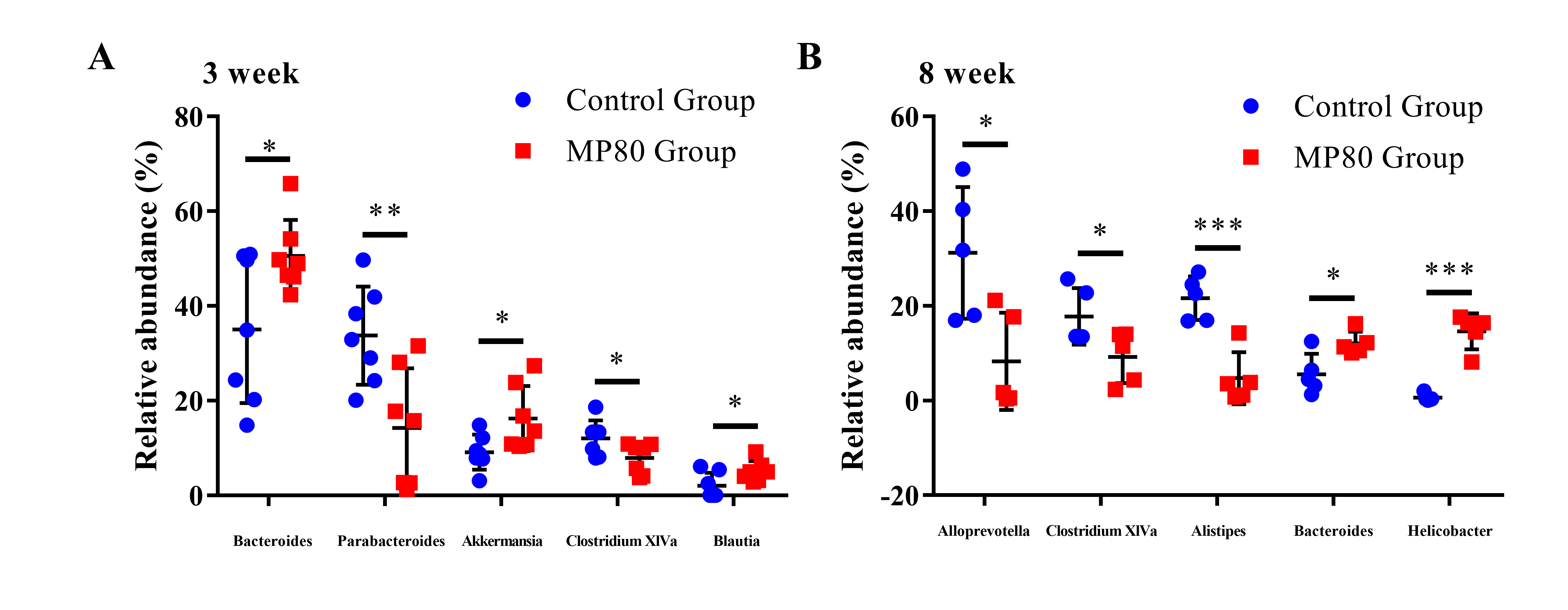

Supplement: FIG S1 [file msystems.01337-20-sf001.tif]
