# Supplementary material for: Maternal Emulsifier P80 Intake Induces Gut Dysbiosis in Offspring and Increases Their Susceptibility to Colitis in Adulthood
Source: mSystems. 2021 Mar 16;6(2):e01337-20. doi: 10.1128/mSystems.01337-20 (PMC8547008; doi:10.1128/mSystems.01337-20)
Supplement: TABLE S1 [file msystems.01337-20-st001.docx]

**Table S1. The Oligonucleotide primers used in Realtime-PCR.**

|  | Primer sequences (5′- 3′) |
| --- | --- |
| GAPDH | Forward primer: GGAGAAACCTGCCAAGTATG |
|  | Reverse primer: TGGGAGTTGCTGTTGAAGTC |
| TNF-α | Forward primer: CTTCTGTCTACTGAACTTCGGG |
|  | Reverse primer: CAGGCTTGTCACTCGAATTTTG |
| IFN-γ  IL-1β  IL-6  ZO-1  CLND-3  APRIL  KC  OCLND  MUC2 | Forward primer: GCATCTTGGCTTTGCAGCT |
|  | Reverse primer: CCTTTTTCGCCTTGCTGTTG  Forward primer: ACGGACCCCAAAAGATGAAG  Reverse primer: TTCTCCACAGCCACAATGAG  Forward primer: CCAGTTGCCTTCTTGGGACT  Reverse primer: GGTCTGTTGGGAGTGGTATCC  Forward primer: GGGCCATCTCAACTCCTGTA  Reverse primer: AGAAGGGCTGACGGGTAAAT  Forward primer: CCTGTGGATGAACTGCGTG  Reverse primer: GTAGTCCTTGCGGTCGTAG  Forward primer: ATGGATTACAAAGACGATGACG  Reverse primer: TCACAGTTTCACA AACCCCAGG  Forward primer: AACCGAAGTCATAGCCACAC  Reverse primer: CAGACGGTGCCATCAGAG  Forward primer: CGGTACAGCAGCAATGGTAA  Reverse primer: CTCCCCACCTGTCGTGTAGT  Forward primer: TCGCCCAAGTCGACACTCA  Reverse primer: GCAAATAGCCATAGTACAGTTACACAGC |
